# Validation of Genes Affecting Rice Grain Zinc Content Through Candidate Gene-Based Association Analysis

**DOI:** 10.3389/fgene.2021.701658

**Published:** 2021-08-09

**Authors:** Jindong Liu, Junhui Zhan, Jingguang Chen, Xiang Lu, Shuai Zhi, Guoyou Ye

**Affiliations:** ^1^Guangdong Laboratory of Lingnan Modern Agriculture, Genome Analysis Laboratory of the Ministry of Agriculture and Rural Affairs, Agricultural Genomics Institute at Shenzhen, Chinese Academy of Agricultural Sciences, Shenzhen, China; ^2^CAAS-IRRI Joint Laboratory for Genomics-Assisted Germplasm Enhancement, Agricultural Genomics Institute at Shenzhen, Chinese Academy of Agricultural Sciences, Shenzhen, China; ^3^School of Agriculture, Sun Yat-sen University, Guangzhou, China; ^4^Strategic Innovation Platform, International Rice Research Institute, Makati, Philippines

**Keywords:** association analysis, grain zinc content (GZC), haplotype, mrMLM, *Oryza sativa* L.

## Abstract

Several key genes governing Zn homeostasis and grain zinc content (GZC) have been functionally characterized. However, the effects of these genes in diverse breeding populations have not been evaluated; thus, their availability in breeding is unclear. In this study, the effects of 65 genes related to rice zinc responses on GZC were evaluated using two panels of breeding lines, and the superior haplotypes were identified. One panel consisted of mega varieties from the International Rice Research Institute (IRRI), South Asia, and Southeast Asia (SEA), and the other panel is breeding lines/varieties from South China (SC). In addition, a multiparent advanced generation intercross (MAGIC) population, named as DC1, was also employed. Three analytical methods, single-locus mixed linear model (SL-MLM), multilocus random-SNP-effect mixed linear model (mrMLM), and haplotype-based association analysis (Hap-AA), were applied. *OsIDEF1* (which explained 12.3% of the phenotypic variance) and *OsZIFL7* (8.3–9.1%), *OsZIP7* (18.9%), and *OsIRT1* (17.9%) were identified by SL-MLM in SEA and SC, respectively, whereas no gene was significantly associated with GZC in DC1. In total, five (*OsNRAMP6*, *OsYSL15*, *OsIRT1*, *OsIDEF1*, and *OsZIFL7*, 7.70–15.39%), three (*OsFRDL1*, *OsIRT1*, and *OsZIP7*, 11.87–17.99%), and two (*OsYSL7* and *OsZIP7*, 9.85–10.57%) genes were detected to be significantly associated with GZC in SEA, SC, and DC1 by mrMLM, respectively. Hap-AA indicated that *Hap1-OsNRAMP5*, *Hap5-OsZIP4*, *Hap1-OsIRT1*, *Hap3-OsNRAMP6*, *Hap6-OsMTP1*, and *Hap6-OsYSL15* had the largest effects for GZC in SEA, whereas *Hap3-OsOPT7*, *Hap4-OsIRT2*, *Hap4-OsZIP7*, *Hap5-OsIRT1*, and *Hap5-OsSAMS1* were the most significant in the SC population. Besides, superior alleles were also identified for the significant genes. The genes significantly associated with GZC and their superior haplotypes identified in different panels could be used in enhancing GZC through molecular breeding, which could further address the problem of Zn malnutrition among rice consumers.

## Introduction

Zinc (Zn) is an important micronutrient for global human nutritional status (Keith et al., 2006; Sadeghzadeh, 2013). Zinc deficiency has been associated with serious health concerns, particularly in children from the developing world. Rice is an important crop for more than a quarter of the global population (Bouis and Welch, 2010; Swamy et al., 2016). Therefore, biofortifying rice grain Zn content is an efficient approach to combat Zn malnutrition. Nevertheless, genetic improvement of grain zinc content (GZC) is relatively cost-effective and efficient compared to the agronomic or postharvest processing method used for Zn biofortification (White and Broadley, 2011; Swamy et al., 2016).

Recent advances in rice functional genomics facilitated the cloning and functional characterization of genes involved in zinc absorption, transport, and accumulation, such as the nicotinamide synthase gene family (*OsNAS1*, *OsNAS2*, and *OsNAS3*), oligopeptide transport gene family (*OsPT2* and *OsYSL15*) (Inoue et al., 2003; Johnson et al., 2011), ZIP gene family (*OsZIP1*, *OsZIP3*, *OsZIP4*, *OsZIP5*, *OsZIP7*, and *OsZIP8*) (Ishimaru et al., 2007; Lee et al., 2010a, b), vacuolar membrane transporters (*OsVIT1* and *OsVIT2*), *OsFER2*, *OsIRT1*, *OsMTP1*, *OsHMA2*, and *OsFRDL1* (Lee and An, 2009; Zhang et al., 2012; Swamy et al., 2016). Several studies have shown that the overexpression of *OsNAS* genes (*OsNAS1*, *OsNAS2*, and *OsNAS3*) improved GZC by several folds (Lee et al., 2010a, b; Johnson et al., 2011). The *ZIP* family genes are important metal transporters involved in Zn transportation within and between different parts of the rice plant (Ramesh et al., 2003; Ishimaru et al., 2007, 2011). Several transcription factor genes such as *OsNAC*, *NAM-B1*, *OsIDEF1*, *OsIDEF2*, and *OsIRO2* were shown to be important regulators of metal homeostasis (Ogo et al., 2008; Waters et al., 2009; Gande et al., 2014; Swamy et al., 2016) and may also regulate Zn deficiency-responsive genes.

The generated genomic resources would pave the way for identification of donors, alleles, and haplotypes associated with traits of interest (Varshney et al., 2009; Alexandrov et al., 2015). The GZC is a typical quantitative trait controlled by many genes individually, explaining tiny proportions of the total observed phenotypic variation (Swamy et al., 2016). Natural variants and haplotype association analysis have been proven to be more beneficial in capturing genes associated with complex traits. Previous approaches for genetic improvement of GZC were mainly based on traditional mapping using populations derived from biparental crosses that hardly consider the existence and effects of natural variants and haplotypes in the populations (Swamy et al., 2016). However, it is noteworthy that only few cloned genes are being used in breeding (Swamy et al., 2016). The details of favorable haplotypes for corresponding genes are crucial for crop breeding (Bevan et al., 2017; Abbai et al., 2019; Sinha et al., 2020). Thus, identifying variations and superior haplotype of genes controlling grain Zn-related traits in diverse panels will provide valuable targets for molecular marker-assisted selection (MAS).

Association analysis is a powerful approach to uncover genetic mechanism for such complex polygenic traits (Flint-Garcia et al., 2003; Zhu et al., 2008). The candidate-gene-based association analysis targets genes within the functional regions of the genome, thereby increasing the resolution to detect significant gene–trait associations (Flint-Garcia et al., 2003; Zhu et al., 2008). For instance, using candidate gene analysis, genes governing complex traits were identified in *Arabidopsis*, wheat, peas, potato, rye, and perennial ryegrass (Zhao et al., 2015). The single-locus association analysis (SL-AA) and single-locus mixed linear model (SL-MLM) are the established analytical methods to detect genetic variants for agronomic traits (Zhu et al., 2008; Cui et al., 2018). The single locus-based approaches are limited in detecting marginal effects of quantitative trait nucleotides (QTNs) influenced by the polygenic background and often requires correction for multiple tests (Zhang et al., 2020). The stringent Bonferroni correction limited the detection of small-effect loci (Wang et al., 2020; Zhang et al., 2020), which may cumulatively explain a significant amount of the observed phenotypic variation. On the other side, a more recent method known as multilocus association analysis (ML-AA) addresses these shortcomings by simultaneously scanning and estimating all the marker effects across the whole genome (Wen et al., 2018; Zhang et al., 2020). ML-AA slightly outperformed single locus-based methods in detecting significant QTNs associated with several complex traits (Tamba and Zhang, 2018; Wen et al., 2018).

Until now, many MAS breeding practices have been performed to disease-, resistance-, and yield-related genes (Wang et al., 2020). However, MAS for higher GZC is limited because of the rare information of genes and their haplotype for deploying MAS. In the present study, candidate gene-based association analysis was used to test the effects of 65 genes (Supplementary Table 1), which have been previously characterized for their influence on rice GZC. Two panels of breeding lines and a multiparent advanced generation intercross (MAGIC) population were used. Three analytical methods including SL-MLM, multilocus random-SNP-effect mixed linear model (mrMLM) model, and haplotype-based association analysis (Hap-AA) were applied to maximize the probability of catching all the important genes, meanwhile controlling the false positives.

## Materials and Methods

### Plant Materials

Two breeding populations from the International Rice Research Institute (IRRI), South Asia, and Southeast Asia (SEA) (Supplementary Table 2) and from South China (SC) (Supplementary Table 3) were used in this study, respectively. The SEA panel including 207 accessions was mainly from IRRI, South Asia, and SEA (Liu et al., 2020), whereas the SC panel including 99 breeding lines/varieties was from SC. In addition to the two breeding populations, a four-parent MAGIC population, named as DC1 (215 lines), previously characterized by Meng et al. (2017), was also evaluated for GZC. The MAGIC-DC1 was developed at IRRI by intercrossing four elite indica founder lines, including (A) SAGC-08, (B) HHZ5-SAL9-Y3-Y1, (C) BP1976B-2-3-7-TB-1-1, and (D) PR33282-B-8-1-1-1-1-1, which shows better grain quality, higher yield potential, and biotic and abiotic stress tolerance (Liu et al., 2021).

### Genotyping, Population Structure, and Haplotype Analysis

Total genomic DNA for genotyping was extracted from young leaves using the Cetyl trimethyl ammonium bromide (CTAB) procedure. The accessions from SEA and SC were genotyped using the Illumina HiSeq 2000 (PE150) (50X) by Berry Genomics Corporation, Beijing, China^1^. Reads were aligned to the Nipponbare RefSeq (IRGSP-1.0)^2^ using BWA-MEM V0.7.10^3^. The duplicated reads were sorted out using Picard tools^4^. The variants for each accession were called by the GATK V3.2.2 best practices^5^. A stringent filtering strategy was conducted to choose high-quality SNPs and InDels for subsequent analysis (QUAL < 30.0, QD < 10.0, FS > 200.0, MQRankSum < −12.5, and ReadPosRankSum < −8.0). Next, the DC1 was sequenced with the 55K Affymetrix Axiom Rice Genotyping Array at the Capital Bio-Technology^6^ (Beijing, China), and the SNP data set was obtained according to Meng et al. (2017). Markers with minor allele frequency (MAF) < 0.05 and missing rate > 0.05 were removed.

The nucleotide variations (SNPs and InDels) from SEA and SC were annotated by ANNOVAR (Wang et al., 2010). The SNPs and Indels located in the CDS and the promoter (−1,500 bp) region of the 65 selected genes were extracted and used for subsequent association and haplotype analysis. Haplotype analysis for all the genes was carried out by considering the non-synonymous SNPs and Indels, which can lead to amino acid change. The correlated markers (*r*^2^ = 1.0) for SNPs were excluded from the genotype data set. Besides, the SNPs were filtered according to the following requirements: (1) only two alleles, (2) exclude SNPs of missing data > 0.9, (3) MAF ≥ 0.05, and (4) mean depth values ≥ 5. The haplotype analysis was conducted by CandiHap V2.0^7^ based on R 4.0.1.

Population structure, principal components analysis (PCA), and neighbor-joining (NJ) tree analysis were used to infer the population structure and kinship for SEA, SC, and DC1. Population structure was analyzed using 10,000 polymorphic SNP markers with Admixture 1.3.0 (Alexander and Lange, 2011). PCA and NJ trees were calculated by the software Tassel v5.1^8^ (Bradbury et al., 2007). The structure and PCA for SEA and DC1 populations have been previously reported by Meng et al. (2017) and Liu et al. (2020), respectively.

### Grain Zn Concentration Measurement

The grain samples from all three populations were dried at 65°C for 3 days. Then, the dried grains were crushed, wet digested in concentrated HNO_3_ at 120°C (30 min), and further digested with HClO_4_ at 180°C until they became transparent. The samples were then diluted with ultrapure water, and the zinc concentrations were evaluated by inductively coupled plasma mass spectrometry (ICP-MS) according to Liu et al. (2020).

### Association Analysis and Superior Haplotype Identification

The SNPs and Indels extracted from the CDS and promoter (−1,500 bp) regions were used to conduct association analysis for SEA and SC populations, whereas the SNPs located in the LD decay interval (150 kb) were selected for association analysis in DC1. Association analysis was carried out using the Tassel V5.1 (Bradbury et al., 2007) with a mixed linear model accounting for both population structure and kinship (Bradbury et al., 2007). The Manhattan and QQ plots were displayed using the R package CMplot^9^. Another R package, mrMLM V2.1, was used to realize the mrMLM algorithm (Wang et al., 2020). The critical threshold of significance for marker–trait association (MTA) was according to Bonferroni correction in SL-MLM and at an Logarithm of odds (LOD) value of 3 in mrMLM. A QTN was defined as a haplotype block possessing SNPs identified as significantly associated with GZC trait. For Hap-AA, false discovery rate < 0.1 was chosen as the significance threshold.

The resultant significant genes were further used to find superior haplotypes by conducting a Duncan analysis of GZC means (haplotype-wise) for each subgroup across SEA and SC panels. Furthermore, to ensure the accuracy of the results, only the haplotypes validated in at least five lines were considered for statistical analysis. In the present study, superior haplotypes were those with significantly higher average GZC (*p* < 0.05) but not the most frequent one in the subgroup.

## Results

### Phenotypic Variation Analysis

Continuous variation was observed for GZC across the SEA, SC, and DC1 panels with approximately normal distributions (Supplementary Figure 1 and Supplementary Tables 2–4). In the SEA panel, the GZC ranged from 4.5 to 68.0 mg/kg with an average of 32.0 mg/kg (Supplementary Table 2). GZC ranged from 9.4 to 26.2 mg/kg, with an average of 16.9 mg/kg (Supplementary Table 3) in the SC panel. Meanwhile, average GZC was 32.8 mg/kg, ranging from 7.5 to 72.5 mg/kg in the DC1 panel. The GZC was highest and lowest in parental lines HHZ 5-SAL9-Y3-Y1 and PR33282-B-8-1-1-1-1-1, respectively (Supplementary Table 6).

### Genotyping

A total of 3,530 SNPs and 153 Indels were identified in the CDS and promoter regions of 65 selected genes in SEA and SC. The SNPs and Indels ranged from 1 to 135 and from 0 to 34, respectively. The average numbers of SNPs and Indels were 53.3 and 2.3 for the 65 selected genes, respectively (Supplementary Table 7 and Supplementary Figure 2). For the DC1, a total of 1,753 SNPs were identified at the Linkage disequilibrium (LD) decay intervals for the selected genes (Supplementary Table 7 and Supplementary Figure 2).

### Population Structure

**FIGURE 1:**
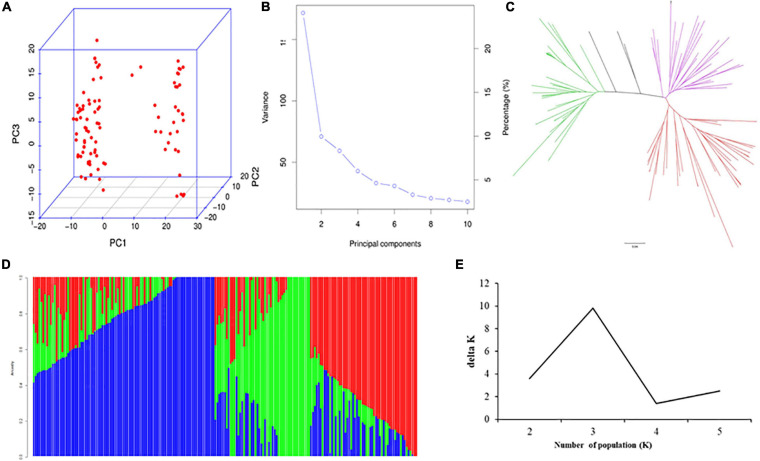
Population analysis for the accessions from the SC panel. **(A)** PCA plots, **(B)** total variation explained by PCs, **(C)** NJ tree, **(D)** three subgroups inferred by structure analysis, **(E)** estimated Δ*K* over five repeats of structure analysis.

According to Liu et al. (2020), the 207 accessions from SEA are divided into two subgroups, the *Japonica* subpopulation (*SEA-Japonica*) and the *Indica* subpopulation (*SEA-Indica*). The characterization of both subpopulations is consistent with their geographic origins. However, some levels of admixture between the *Indica* and *Japonica* subpopulations were detected in this study. The *SEA-Indica* accessions included the mega varieties from IRRI, South Asia, and SEA, and few released cultivars were from SC. On the other side, most of the accessions from the *SEA-Japonica* subpopulation were from the SC panel. Structure, PCA, and NJ tree analysis of the SC panel divided the 99 indica accessions into three subgroups, namely, *SC-Indica1*, *SC-Indica2*, and *SC-Indica3* (Figure 1). The *SC-Indica1* accessions included several landraces and other cultivars, which were released around the 1960s to 1980s (e.g., Dasuikuai, Jiuzhan, and Qinghuaai 6). The *SC-Indica2* accessions mainly included cultivars released from the 1960s to 1990s (e.g., Guangchangai 6, Qingguaiai 5, and Aizhenzan). Lastly, majority of the accessions from the *SEA-Indica3* subgroup were cultivars released from Guangdong and Guangxi provinces of China around the 1990s to 2000s, such as shanyou 836-1, Xiangsimiao 2, and Yuanzhen 397. Because of the multiple hybridizations and selfing that were used in developing the DC1 population, no strong population structure was found in this population, as also previously reported by Meng et al. (2017). The total variations of population structure explained by the top three PCs were 28.5, 8.2, and 3.2% (SEA panel); 24.5, 9.2, and 7.3% (SC panel); and 5.9, 5.3, and 4.1% (DC1 panel).

### Haplotype Analysis

Haplotype analysis was conducted for all the 65 selected genes in both the SEA and SC panels. In total, the number of haplotypes for each gene ranged from 3 to 11, while mainly including four to nine haplotypes (57 genes). The largest number of haplotypes was 10 and was recorded for *OsZIFL10* and *OsIRO2*. Further, *OsZIFL2*, *OsOPT1*, and *NRAMP6* genes had only three haplotypes each. The frequency of each haplotypes for the selected genes ranged from 3.25 to 81.2% in all accessions. The range of haplotype frequency was 5.25–72.6% and 6.58–81.6% in SEA and SC, respectively. The highest haplotype frequencies in SEA were recorded in *Hap3-ZIP7* (94.5%), *Hap3-OsZIFL2* (93.2%), *Hap3-VIT1* (93.2%), *Hap3-OsNAAT4* (93.7%), *Hap44-OsOPT4* (92.5%), and *Hap7-OsIDEF2* (85.7%). In the SC panel, *Hap1-OsVIT2* (96.1%), *Hap1-OsZIP5* (95.1%), *Hap1-OsFER1* (94.2%), *Hap1-OsYSL1* (93.2%), *Hap1-OsZIP9* (92.2%), *Hap1-OsZIP4* (91.4%), and *Hap1-OsOPT5* (89.3%) showed the highest frequencies. Furthermore, differences in haplotype frequencies were detectable within each subgroup of SEA and SC panels. In the SEA panel, haplotypes with the highest frequencies were *Hap1-OsZIP6* (94.3%), *Hap1-OsFER1* (96.0%), *Hap1-MTA1* (94.3%), *Hap1-OsNAS3* (96.0%), and *Hap3-OsZIP7* (96.0%), which were found in the *SEA-Japonica* subpopulation, whereas *Hap1-OsVIT2* (93.3%), *Hap1-OsYSL1* (93.3%), *Hap1-OsMTA1* (91.1), *Hap3-OsNAAT4* (91.1%), and *Hap3-OsZIP3* (91.1%) were from the *SEA-Indica*. Haplotypes with the lowest frequencies were *Hap1-OsMTP1* (1.3%), *Hap1-OsFRO2* (1.3%), *Hap2-OsYSL14* (1.3%), *Hap5-OsYSL18* (1.3%), and *Hap6-OsYSL12* (1.3%) from the *SEA-Japonica* subpopulation, and *Hap1-OsNAAT4* (2.2%), *Hap1-OsZIP3* (2.2%), *Hap1-ZIP7* (2.2%), *Hap4-OsNAAT1* (2.2%), and *Hap4-IDEF1* (2.2%) from *SEA-Indica*. Higher haplotype frequencies for the *SC-Indica1* subpopulation of the SC panel were *Hap3-OsZIFL2* (94.4%), *Hap3-OsZIP8* (94.4%), *Hap5-OsNAAT1* (90.5%), *Hap5-OsSAMS1* (90.5%), and *Hap5-OsYSL4* (88.5%). For the *SC-Indica2* of the SC panel, recorded higher haplotype frequencies were *Hap1-OsOPT1* (94.2%), *Hap1-OsZIP49* (94.2%), *Hap3-OsNRAMP4* (92.5%), *Hap3-OsZIP8* (92.5%), and *Hap4-OsZIP9* (88.5%). The higher haplotype distribution frequencies—*Hap1-OsZIP49* (94.5%), *Hap3-OsZIP3* (90.5%), *Hap3-OsYSL4* (90.5%), *Hap5-OsZIP9* (88.2%), and *Hap4-OsVIT2* (88.2%)—were from the *SC-Indica3* population. On the other side, the lowest haplotype frequencies were recorded for *Hap2-OsZIFL2* (5.0%), *Hap4-OsZIP49* (5.0%), *Hap4-OsZIP8* (5.0%), *Hap1-OsZIP3* (5.0%), and *Hap2-OsSAMS1* (5.0%) in the *SC-Indica1* subpopulation. *Hap3-OsOPT1* (3.5%), *Hap4-OsZIP49* (3.5%), *Hap5-OsZIP8* (5.2%), *Hap1-OsZIP3* (5.2%), and *Hap4-OsVIT2* (5.2%) were found in the *SC-Indica2*. Furthermore, *Hap2-OsNAAT1* (2.6%), *Hap8-OsSAMS1* (2.6%), *Hap3-OsZIP6* (4.2%), *Hap2-OsOPT3* (4.2%), and *Hap4-OsFER1* (4.2%) from the *SC-Indica3* subpopulation also showed the lowest frequencies (Supplementary Table 4).

### SL-AA and ML-AA Analyses

**FIGURE 2:**
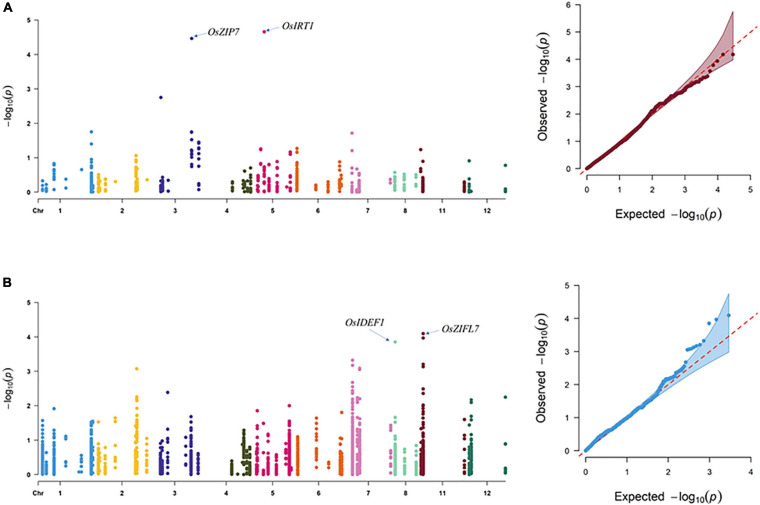
Association analysis for grain Zn content by SL-MLM in the SEA and SC panels. **(A)** SEA panel and **(B)** SC panel.

**FIGURE 3:**
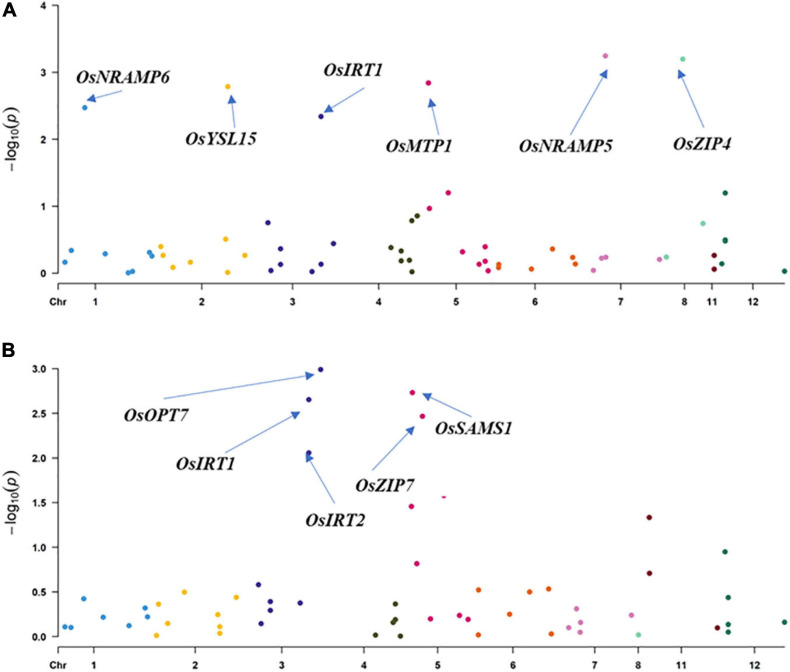
Association analysis for grain Zn content by Hap-GWAS in SEA and SC panel. **(A)** SEA panel and **(B)** SC panel.

In SEA, three SNPs corresponding to *OsIDEF1* and *OsZIFL7* were found to be significantly associated with GZC by SL-MLM, and each explained the phenotypic variation of 12.3 and 8.3–9.1%, respectively (Figure 2 and Table 1). Besides, five significant QTNs (LOD ≥ 3) corresponding to three genes (*OsYSL15*, *OsIDEF1*, and *OsZIFL7*) were simultaneously found to be associated with the GZC by mrMLM in the SEA panel. Each of the five QTNs explained phenotypic variation ranging from 7.70 to 15.39% (Figure 3 and Table 2). As shown by SL-MLM, *OsZIP7* (one SNP) and *OsIRT1* (four SNPs) were significantly associated with GZC in the SC panel and explained phenotypic variations of 18.9 and 17.9%, respectively (Figure 2 and Table 1). Besides, mrMLM showed that three significant QTNs (LOD ≥ 3) corresponding to three genes (*OsFRDL1*, *OsIRT1*, and *OsZIP7*) were significantly associated with GZC in the SC panel and explained a phenotypic variation of 16.08% (*OsFRDL1*), 17.99% (*OsIRT1*), and 11.87% (*OsZIP7*), respectively (Figure 3 and Table 2). In the DC1 panel, no significant GZC genes were identified with SL-MLM. However, mrMLM detected two significant QTNs (LOD ≥ 3) corresponding to two genes (*OsYSL7* and *OsZIP7*). The amounts of phenotypic variation defined by these two genes were 9.85% (*OsYSL7*) and 10.87% (*OsZIP7*), respectively (Figure 3 and Table 2).

**TABLE 1:** List of detected grain Zn content-associated genes in SEA and SC panels based on the SL-MLM model.

Population	Gene name	LOC_ID	Chromosome	Start (bp)	End (bp)	Position (bp)	*p*-value	PVE (%)
SEA	*OsIDEF1*	*LOC_Os08g01090*	8	63574	67839	66241	3.2E-06	12.3%
SEA	*OsZIFL7*	*LOC_Os11g04104*	11	1657863	1668362	1666609	1.1E-04	9.1%
SEA	*OsZIFL7*	*LOC_Os11g04104*	11	1657863	1668362	1666604	2.4E-04	8.3%
SC	*OsZIP7*	*LOC_Os05g10940*	5	6090801	6094142	6092048	2.20E-05	18.9%
SC	*OsIRT1*	*LOC_Os03g46470*	3	26286156	2.6E+07	26287332	3.44E-05	17.9%
SC	*OsIRT1*	*LOC_Os03g46470*	3	26286156	2.6E+07	26290614	3.44E-05	17.9%
SC	*OsIRT1*	*LOC_Os03g46470*	3	26286156	2.6E+07	26290620	3.44E-05	17.9%
SC	*OsIRT1*	*LOC_Os03g46470*	3	26286156	2.6E+07	26291879	3.44E-05	17.9%

**TABLE 2:** List of detected grain Zn content-associated genes in all the panels based on the mrMLM model.

Population	Chromosome	Gene name	LOC_ID	Gene_start (bp)	Gene_end (bp)	Marker position (bp)	LOD score	−log10(*p*)	*r*^2^ (%)
SEA	1	*OsNRAMP6*	*LOC_Os01g31870*	17457002	17465049	17458376	2.90	3.28	7.70
SEA	2	*OsYSL15*	*LOC_Os02g43410*	26200013	26204720	26204377	3.15	3.86	8.49
SEA	3	*OsIRT1*	*LOC_Os03g46470*	26286156	26292023	26276494	5.26	6.07	14.99
SEA	8	*OsIDEF1*	*LOC_Os08g01090*	63574	67839	66241	5.42	6.23	15.39
SEA	11	*OsZIFL7*	*LOC_Os11g04104*	1657863	1668362	1622889	4.92	5.71	11.06
SC	3	*OsFRDL1*	*LOC_Os03g11734*	6131845	6142781	6142106	5.25	6.06	16.08
SC	3	*OsIRT1*	*LOC_Os03g46470*	26286156	26292023	26276494	5.26	6.07	17.99
SC	5	*OsZIP7*	*LOC_Os05g10940*	6090801	6094142	6092048	3.17	3.88	11.87
DC1	2	*OsYSL7*	*LOC_Os02g02450*	862272	865065	760849	3.27	3.68	9.85
DC1	5	*OsZIP7*	*LOC_Os05g10940*	6090801	6094142	6092048	3.42	3.97	10.57

### Association Analysis for Haplotype and Identification of Superior Haplotypes

Hap-AA showed that *OsNRAMP5*, *OsZIP4*, *OsNRAMP6*, *OsMTP1*, *OsYSL15*, and *OsIRT1* are significantly associated with GZC in the SEA panel. The total explained phenotypic variation in the SEA panel ranged from 3.9 to 12.9%. The haplotype variations of *OsOPT7*, *OsIRT1*, *OsSAMS1*, *OsIRT2*, and *OsZIP7* were found to have significant associations with the GZC in SC, and each explained phenotypic variation from 9.6 to 13.6%. Furthermore, the study revealed performance differences among haplotypes. The best haplotypes for GZC were *Hap1-OsNRAMP5* (12.9%), *Hap5-OsZIP4* (11.8%), *Hap3-OsNRAMP6* (6.3%), *Hap6-OsMTP1* (6.1%), *Hap1-OsIRT1* (5.9%), and *Hap6-OsYSL15* (3.9%). In the SC panel, the superior haplotypes for GZC were *Hap3-OsOPT7* (13.8%) *Hap4-OsIRT2* (13.6%), *OsSAMS1* (12.6%), *Hap5-OsIRT1* (12.1%), and *Hap4-OsZIP7* (9.6%) (Figure 3 and Table 3).

**TABLE 3:** List of grain Zn content-associated genes and superior alleles in SEA and SC panels based on haplotype genome-wide association analysis.

Population	Gene	ID	*p*-value	*R*^2^ (%)	Subgroup-1			Subgroup-2			Subgroup-3		
					Superior haplotype	Percentage (%)	Mean GZC (mg/kg)	Superior haplotype	Percentage (%)	Mean GZC (mg/kg)	Superior haplotype	Percentage (%)	Mean GZC (mg/kg)
SEA	*OsNRAMP5*	*LOC_Os07g15370*	3.2 × 10^–4^	12.9	Hap3	6.25	43.2	Hap3	7.89	44.8	–	–	–
SEA	*OsZIP4*	*LOC_Os08g10630*	3.1 × 10^–4^	11.8	Hap2	8.59	50.0	–	–	–	–	–	–
SEA	*OsNRAMP6*	*LOC_Os01g31870*	2.6 × 10^–3^	6.3	Hap3	19.53	45.1	Hap3	34.90	43.2	–	–	–
SEA	*OsMTP1*	*LOC_Os05g03780*	2.9 × 10^–3^	6.1	Hap7	5.46	47.8	Hap6	13.15	41.3	–	–	–
SEA	*OsYSL15*	*LOC_Os02g43410*	2.7 × 10^–3^	3.9	–	–	–	–	–	–	–	–	–
SEA	*OsIRT1*	*LOC_Os03g46470*	2.5 × 10^–3^	5.9	Hap8	2.34	47.4	Hap1	21.50	33.5	–	–	–
SC	*OsOPT7*	*LOC_Os03g54000*	2.9 × 10^–3^	13.8	–	–	–	Hap2	20.10	18.5	Hap2	22.50	18.2
SC	*OsIRT1*	*LOC_Os03g46470*	2.7 × 10^–3^	12.1	–	–	–	Hap5	15.20	20.7	Hap5	20.00	19.6
SC	*OsSAMS1*	*LOC_Os05g04510*	2.9 × 10^–3^	12.6	–	–	–	–	–	–	–	–	–
SC	*OsIRT2*	*LOC_Os03g46454*	2.1 × 10^–3^	13.6	–	–	–	Hap4	8.90	18.4	Hap4	20.20	20.2
SC	*OsZIP7*	*LOC_Os05g10940*	2.6 × 10^–3^	9.6	Hap1	8.50	16.2	Hap4	12.80	43.2	Hap4	20.00	21.2

To reduce noises from the structure analysis, the Duncan’s test was established to identify the superior haplotypes from the significant genes identified by Hap-AA for each subgroup in the SEA and SC panels. Five superior haplotypes were identified in the *SEA-Indica* subgroup, including *Hap3-OsNRAMP5* (6.25%, 43.2 mg/kg, and 27.2–36.3 mg/kg for other haplotypes), *Hap2-OsZIP4* (8.59%, 50.0 mg/kg, and 33.5–41.0 mg/kg for other haplotypes), *Hap3-OsNRAMP6* (19.53%, 45.1 mg/kg, and 30.5–36.9 mg/kg for other haplotypes), *Hap7-OsMTP1* (5.46%, 47.8 mg/kg, and 27.2–36.2 mg/kg for other haplotypes), and *Hap8-OsIRT1* (2.34%, 47.4 mg/kg, and 26.8–32.1 mg/kg for other haplotypes); four superior haplotypes were identified in *SEA-Japonica*, including *Hap3-OsNRAMP5* (7.89%, 44.8 mg/kg, and 32.3–35.7 mg/kg for other haplotypes), *Hap3-OsNRAMP6* (34.9%, 43.2 mg/kg, and 27.6–36.9 mg/kg for other haplotypes), *Hap6-OsMTP1* (13.15%, 41.3 mg/kg, and 28.3–35.5 mg/kg for other haplotypes), and *Hap1-OsYSL15* (21.5%, 33.5 mg/kg, and 25.2–29.4 mg/kg for other haplotypes mg/kg) (Supplementary Tables). Likewise, *Hap1-OsZIP7* (8.5%, 16.2 mg/kg, and 13.5–14.8 mg/kg for other haplotypes) for GZC was detected as a superior haplotype in *SC-Indica1*; four superior haplotypes were detected in *SC-Indica2*, including *Hap2-OsOPT7* (20.1%, 18.5 mg/kg, and 15.6–20.2 mg/kg for other haplotypes), *Hap5-IRT1* (15.2%, 20.7 mg/kg, and 12.8–14.3 mg/kg for other haplotypes), *Hap4-OsIRT2* (8.9%, 18.4 mg/kg, and 14.5–17.2 mg/kg for other haplotypes), and *Hap4-ZIP7* (12.8%, 17.2 mg/kg, and 8.2–16.0 mg/kg for other haplotypes); another four superior haplotypes were detected in *SC-Indica3*, including *Hap2-OsOPT7* (22.5%, 18.2 mg/kg, and 14.2–15.6 mg/kg for other haplotypes), *Hap5-IRT1* (20.0%, 19.6 mg/kg, and 13.6–17.8 mg/kg for other haplotypes), *Hap4-OsIRT2* (12.5%, 20.2 mg/kg, and 15.8–18.9 mg/kg for other haplotypes), and *Hap4-ZIP7* (20.0%, 21.2 mg/kg, and 15.5–18.9 mg/kg for other haplotypes) (Table 3).

## Discussion

Although dozens of Zn response genes have been cloned and validated, their usefulness in breeding remains unclear (Rose et al., 2013; Swamy et al., 2016). Deeper insights into the complex relationship among GZC and genes would greatly aid in the selection of appropriate genes and haplotypes to enhance Zn biofortification in rice. In the present study, associations of GZC with variations and haplotype of selected genes were separately conducted to find genes and their superior haplotypes.

The distributions of haplotypes were different in the SEA and SC panels. Previous studies have also reported that haplotype distributions differ across populations (Qian et al., 2017; Wang et al., 2019). In the present study, *Hap1-OsZIFL2*, *Hap3-OsZIP49*, *Hap2-OsNRAMP4*, *Hap3-OsZIP8*, and *Hap3-OsNAAT1* were only identified in the SEA panel, whereas *Hap4-OsZIFL12* and *Hap7-OsNRAMP8* were only detected in the SC panel. The frequencies of haplotype distribution in the SEA and SC panels were also different. The *Hap3-OsOPT1*, *Hap3-OsNAAT1*, and *Hap2-OsNRAMP5* accounted for 40.5, 23.9, and 48.2% of total GZC content variation in the SEA panel, respectively. Yet, these haplotypes only accounted for 17.2% (*Hap3-OsOPT1*), 0 (*Hap3-OsNAAT1*), and 6.0% (*Hap2-OsNRAMP5*) in the SC panel, respectively. Similar findings were also observed for *OsFRDL1*, *OZIFL12*, and *OsNRAMP1*. We also observed that differences in haplotype frequencies within subgroups of the SEA and SC panels. The *Hap1-OsOPT1* with 71.2% of haplotype frequency was the major haplotype in the *SEA-Indica* subgroup, while it only accounted for 23.5% in the *SEA-Japonica* subgroup. The frequencies of *Hap1-OsNRAMP4* and *Hap1-OsYSL14* were 53.0 and 56.0% in the *SEA-Indica* subgroup, respectively. However, in the *SEA-Japonica* subgroup, the haplotype frequencies were 4.2% (*Hap1-OsNRAMP4*) and 18.2% (*Hap1-OsYSL14*). In the SC panel, the haplotype frequencies of *Hap1-OsYSL14* was 70.0% (*SC-Indica1* subgroup), 46.7% (*SC-Indica3*), and 23.5% (*SC-Indica2*); the *Hap1-DMAS1* accounted for 35.0% in *SC-Indica1* but was not found in *SC-Indica2* and *SC-Indica3*. Thus, identifying the genes significantly associated with GZC and its superior haplotypes by Hap-AA is crucial for rice breeding because of the various differences of haplotype distribution.

Among the genes significantly associated with GZC, *OsIRT1*, and *OsZIP7* were detected in both the SEA and SC panels. This imply that these genes play a stabilizing role in diverse accessions and could be widely used in rice breeding. Besides, *OsIDEF1*, *OsIRT1*, and *OsZIP7* genes explained the highest phenotypic variations in the SEA, SC, and DC1 panels, respectively. *OsIDEF1* involved in Zn synthesis and metabolism could be used for Zn accumulation by upregulating the expression of metal homeostasis genes (Ogo et al., 2008; Ishimaru et al., 2011; Swamy et al., 2016). *OsIRT1* and *OsZIP7* are involved in Zn uptake and translocation in plants and can be used for enhancing micronutrient levels in grains (Lee and An, 2009; Swamy et al., 2016). *OsNRAMP6* (SEA panel), *OsYSL15* (SEA panel), *OsZIFL7* (SC panel), *OsFRDL1* (SC panel), and *OsYSL7* (DC1 panel) are also significantly associated with GZC. Several studies have demonstrated that OsYSL family proteins are involved in the pathway of phloem transport and long-distance transport of metals (Inoue et al., 2009; Kakei et al., 2012). *OsYSL15* and *OsYSL7* are iron-regulated iron (III)-deoxymugineic acid transporters expressed in the root tissues and essential for Zn uptake in rice seedlings (Inoue et al., 2009; Lee and An, 2009; Sperotto et al., 2010). *ZINC-INDUCED FACILITATOR-LIKE* (*ZIFL*) family genes are essential for grain Fe and Zn accumulation. It was shown that *ZIFL* genes interact with *OsSPL14* and *OsSPL16* to increase grain yield (Swamy et al., 2018). *OsZIFL7* is a crucial metallic element transporter Zn homeostasis gene. The loss-of-function *OsZIFL7* mutant has altered Zn distribution. Also, the transcription of *OsZIFL7* is upregulated by Zn excess (Ricachenevsky et al., 2011). Both *OsNRAMP5* and *OsNRAMP6* are important for Zn transport and accumulation (Sasaki et al., 2012; Swamy et al., 2016). Li et al. (2014) reported that *OsFRDL1* significantly increased Zn content in the wild type compared to mutants with *OsFRDL1* loss of function. *OsZIP4* and *OsMTP1* were significantly associated with GZC in the SEA panel as shown by Hap-AA. Meanwhile, the haplotype variations of *OsOPT7*, *OsSAMS1*, and *OsIRT2* were found to have significant associations with GZC in SC. *OsMTP1* is a metal tolerance protein (MTP) family gene with Zn transport functions in plants. It localizes at the tonoplast and plays crucial roles in Zn accumulation (Dräger et al., 2004; Yuan et al., 2012; Swamy et al., 2016). Like *OsIRT1*, *OsIRT2* is responsible for Zn uptake from soil, translocation distribution, and redistribution in root and shoot. *OsIRT2* is also essential for grain Zn storage in rice (Ramesh et al., 2003; Lee et al., 2010a, b). *OsNAS*, *OsOPT*, and *OsSAMS* are involved in the biosynthesis, transport, and secretion of phytosiderophores in the root zone, thereby increasing Zn uptake in roots (Bashir et al., 2006; Inoue et al., 2008; Johnson et al., 2011; Swamy et al., 2016).

Although some significant genes were related with high Phenotypic variation explained (PVE), some were related with low GZC in several lines. This may be due to the following reasons: firstly, each gene contains multiple haplotypes, ranging from 3 to 12 in our study. Although the superior haplotypes are often with high GZC, there was no significant difference for most of the other haplotypes. Also, the difference of the haplotypes and GZC caused significant PVE. Besides, GZC is a typical complex trait and controlled by numerous genes. Until now, over 60 genes and 100 loci have been reported associated with GZC. Previous studies have reported the interaction among the genes related to Zn homeostasis and GZC (Swamy et al., 2016).

The conventional single-locus methods have been widely implemented to identify genetic variants in many cereals (Liu et al., 2017; Liu et al., 2020). However, these models have certain shortcomings as they ignore the overall effects of multiple loci and thus suffer from multiple test corrections for critical values (Wang et al., 2020; Zhang et al., 2020). Differing from the single-locus methods, mrMLM is a two-stage method. In this method, the SNP effect is viewed as being random, and all the potentially associated markers are selected by a random-SNP-effect MLM with a modified Bonferroni correction for significance test. At the second stage, all the selected markers are placed into one model, and all the non-zero effects are further detected by a likelihood ratio test for QTN identification (Wen et al., 2018; Zhang et al., 2020). In the present study, mrMLM outperformed the SL-MLM for the number of significant QTNs detected in both the SEA and SC panels. Genes that play crucial roles in plant growth and development such as *OsYSL15*, *OsIRT1*, *OsZIFL3*, and *OsFRDL1* were not detected by the SL-MLM method (Inoue et al., 2009; Lee and An, 2009; Swamy et al., 2016). These data illustrate that the mrMLM approach is more effective and powerful to detect small-effect QTNs from complex traits (Segura et al., 2012; Cui et al., 2018; Wen et al., 2018; Zhang et al., 2020). One potential reason is that the mrMLM method improves the power and accuracy for QTN detection due to the nature of the statistical model (Zhang et al., 2020). Another possible reason was the relatively stringent threshold inherent with the SL-MLM method. For both the SEA and SC panels, most of the loci identified in Hap-AA were also observed with both the SL-MLM and mrMLM methods. These loci were, for instance, *OsNRAMP6*, *OsYSL15*, and *OsIRT1* from the SEA panel, and *OsIRT1* and *OsZIP7* from the SC panel. Furthermore, several genes that were not identified in SL-MLM and mrMLM could be identified with the Hap-AA method. These were *OsNRAMP5*, *OsZIP4*, and *OsMTP1* (SEA panel), and *OsOPT7*, *OsSAMS1*, and *OsIRT2* (SC panel). Likewise, *OsIDEF1* and *OsZIFL7* (SEA panel) and *OsFRDL1* (SC panel) loci identified with SL-MLM and mrMLM could not be identified with the Hap-AA. Based on the above analysis, Hap-AA is an effective and reliable approach for genetic analysis of complex traits. The various methods lead to the differences observed in results. We believe that the results should be interpreted according to the purpose of the study. In this study, we want to provide more potential genes for rice breeding. Thus, we analyzed all the genes found in these methods. However, if we want to identify new genes and clone them, the genes shared by all the three methods may be the best. In summary, combining the merits of SL-MLM, mrMLM, and Hap-AA methods is an effective approach to uncover the genetic architecture for complex agronomic traits (Wu et al., 2016; Zhang et al., 2020).

The use of certain germplasms with higher Zn as donors and an elite line as recurrent parents is commonly adopted in biofortification breeding (Sinha et al., 2020). Normally, at least two steps should be taken during parental selection. Firstly, the existing haplotypes of the target genes in the recurrent parents and donors should be clarified by genotyping and haplotype analysis. Secondly, the favorable alleles/haplotypes transferred from donors to the recurrent parents need to be determined. In the present study, *OsIRT1* and *OsZIP7* were identified in two unique breeding populations and play a stabilizing role in diverse accessions. In the high-GZC breeding progresses of SC and SEA, *OsIRT1* and *OsZIP7* should be considered.

As the distribution of favorable genes and superior haplotypes is different, different genes and haplotypes should be selected from different regions. According to the results from the association analysis, *OsIDEF1* (explained 12.3% of the phenotypic variance) and *OsZIFL7* (8.3–9.1%), *OsZIP7* (18.9%), and *OsIRT1* (17.9%) were identified by SL-MLM in SEA and SC, respectively. In total, five (*OsNRAMP6*, *OsYSL15*, *OsIRT1*, *OsIDEF1*, and *OsZIFL7*, 7.70–15.39%), three (*OsFRDL1*, *OsIRT1*, and *OsZIP7*, 11.87–17.99%), and two (*OsYSL7* and *OsZIP7*, 9.85–10.57%) genes were detected significantly associated with GZC in SEA, SC, and DC1 by mrMLM, respectively. Hap-AA indicated that *OsNRAMP5*, *OsZIP4*, *OsIRT1*, *OsNRAMP6*, *OsMTP1*, and *OsYSL15* had the largest effects for GZC in SEA, whereas *OsOPT7*, *OsIRT2*, *OsZIP7*, *OsIRT1*, and *OsSAMS1* were the most significant in the SC population. Of these, *OsIRT1* and *OsIRT2* significantly associated with GZC in both the SEA and SC panels, whereas *OsZIP7* plays crucial roles for GZC in both the SC and DC1 panels. *OsNRAMP5*, *OsZIP4*, *OsMTP1*, *OsYSL15*, *OsNRAMP6*, *OsIDEF1*, and *OsZIFL7* significantly associated with GZC in SEA; *OsSAMS1*, *OsOPT7*, and *OsFRDL1* were only identified in SC; and *OsYSL7* was detected in DC1. The above results indicated that *OsIRT1* and *OsIRT2* could be considered as target genes in MAS breeding in both SEA and SC; *OsNRAMP5*, *OsZIP4*, *OsMTP1*, *OsYSL15*, *OsNRAMP6*, *OsIDEF1*, and *OsZIFL7* could be used in GZC MAS breeding in SEA; and *OsSAMS1*, *OsOPT7*, and *OsFRDL1* played crucial roles in SC.

*OsYSL15*, *OsIDEF1*, and *OsZIFL7* should be applied in the SEA, whereas *OsFRDL1*, *OsIRT1*, and *OsZIP7* is first choice in SC. *OsZIP7* was also identified in the MAGIC-DC1 panel. This indicates high stability of *OsZIP7* across different breeding populations. Furthermore, superior haplotypes from different subgroups can be selected to improve the GZC of different accessions. The SEA and South Asia regions are major rice production areas. The accessions from the SEA are important germplasm resources for rice breeding. Among the *SEA-Indica* accessions, the *Hap3-OsNRAMP5*, *Hap2-OsZIP4*, *Hap3-OsNRAMP6*, *Hap7-OsMTP1*, and *Hap8-OsIRT1* haplotypes are recommended for GZC improvement. Whereas for the cultivars from the *SEA-Japonica* subgroup, *Hap3-OsNRAMP5*, *Hap3-OsNRAMP6*, *Hap6-OsMTP1*, and *Hap1-OsYSL15* are the best candidate genes and haplotypes. Most of the cultivars from SC, particularly Guangdong province, originated from the traditional breeding methods. For the accessions from the *SC-Indica1* subgroup, *Hap1-OsZIP7* is the first choice; for the *SC-Indica2* accessions, *Hap2-OsOPT7*, *Hap5-IRT1*, *Hap4-OsIRT2*, and *Hap4-ZIP7* haplotypes are the most valuable for GZC improvement; and for the *SC-Indica3* subgroup, priority genes and haplotypes are *Hap2-OsOPT7*, *Hap5-IRT1*, *Hap4-OsIRT2*, and *Hap4-ZIP7.* In summary, the superior haplotypes identified in this study for each subgroup would provide reliable guidelines for future breeding. Lines carrying multiple superior haplotypes, such as IR64, IR72, and IR36 in SEA, Guangchangai 6, Sanhuangzhan 2, and Shuangai 11 can be used to rapidly combine several superior target haplotypes into one background.

## Conclusion

In the present study, 65 grain Zn-related genes were selected to evaluate their effects and identify superior haplotypes in three different populations. In total, seven unique genes were identified for GZC in various populations. Of these, *OsIRT1* and *OsZIP7* were identified in two populations. Also, different superior haplotypes for GZC were identified in the SEA and SC panels. Introgression of these superior haplotypes by the haplotype-based breeding is a promising strategy. This study used robust populations and analytical approaches to identify superior genes and haplotypes, which may pave the way for future breeding efforts for grain Zn content in rice.

## Data Availability Statement

The original contributions presented in the study are included in the article/Supplementary Material, further inquiries can be directed to the corresponding author.

## Author Contributions

JL and JC designed the research, analyzed the physiology data, and drafted the manuscript. JZ, XL, and SZ performed the experiments. GY revised the manuscript. All authors have read, edited, and approved the current version of the manuscript.

## Conflict of Interest

The authors declare that the research was conducted in the absence of any commercial or financial relationships that could be construed as a potential conflict of interest.

## Publisher’s Note

All claims expressed in this article are solely those of the authors and do not necessarily represent those of their affiliated organizations, or those of the publisher, the editors and the reviewers. Any product that may be evaluated in this article, or claim that may be made by its manufacturer, is not guaranteed or endorsed by the publisher.

## Footnotes

1
https://www.berrygenomics.com/

2
http://rice.plantbiology.msu.edu/index.shtml

3
http://bio-bwa.sourceforge.net/bwa.shtml

4
http://broadinstitute.github.io/picard/

5
https://gatk.broadinstitute.org/hc/en-us

6
http://cn.capitalbio.com/

7
https://github.com/xukaili/CandiHap

8
http://www.maizegenetics.net

9
https://github.com/YinLiLin/CMplot

## Supplementary Material

Supplementary Figure 1 Grain Zn grain distribution in the SEA, SC, and DC1 panels.

Supplementary Figure 2 Marker distribution in the SEA, SC, and DC1 panels. **(A)** SNP and InDels in the SEA and SC panels. **(B)** SNP in the DC1 panel.

Supplementary Table 1 List of validated grain Zn content genes used in this study.

Supplementary Table 2 The accessions originated from the SEA panel and haplotype information for grain Zn content-related genes.

Supplementary Table 3 Haplotype information for grain Zn content genes found in the SC cultivars.

Supplementary Table 4 Distribution of haplotype frequencies for the 65 selected zinc responsive genes.

Supplementary Table 5 List of MAGIC-DC1 lines used in this study.

Supplementary Table 6 Parental lines used to develop DC1 MAGIC-DC1 populations.

Supplementary Table 7 Information on SNPs and Indels from the SEA, SC, and DC1 panels.
