# Supplementary material for: Validation of Genes Affecting Rice Grain Zinc Content Through Candidate Gene-Based Association Analysis
Source: Front Genet. 2021 Aug 9;12:701658. doi: 10.3389/fgene.2021.701658 (PMC8381382; doi:10.3389/fgene.2021.701658)
Supplement: Supplementary Figure 1 — Grain Zn grain distribution in the SEA, SC, and DC1 panels. [file Data_Sheet_2.docx]

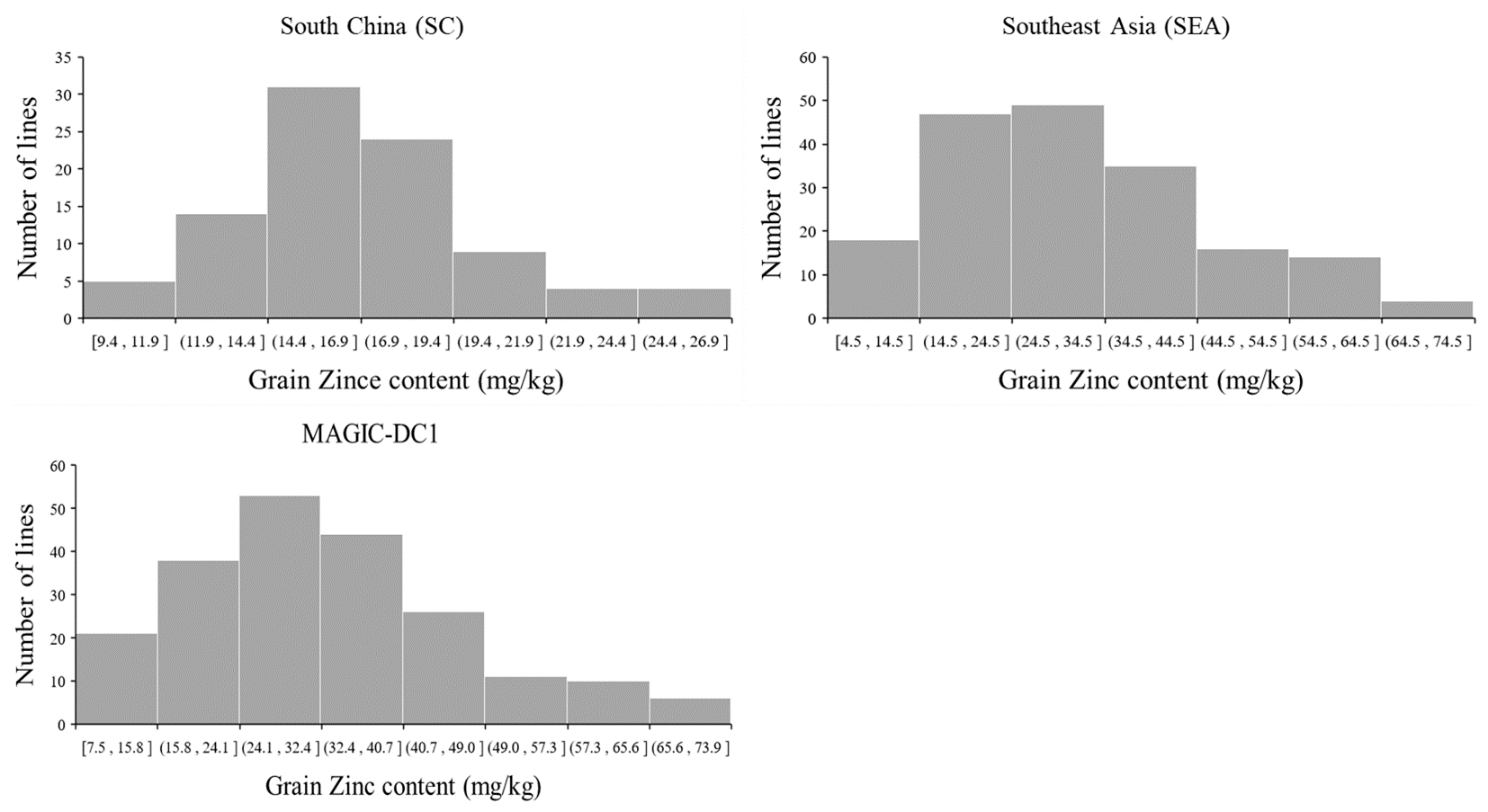


**Figure S1** grain Zn grain distribution in SEA, SC and DC1 panels.


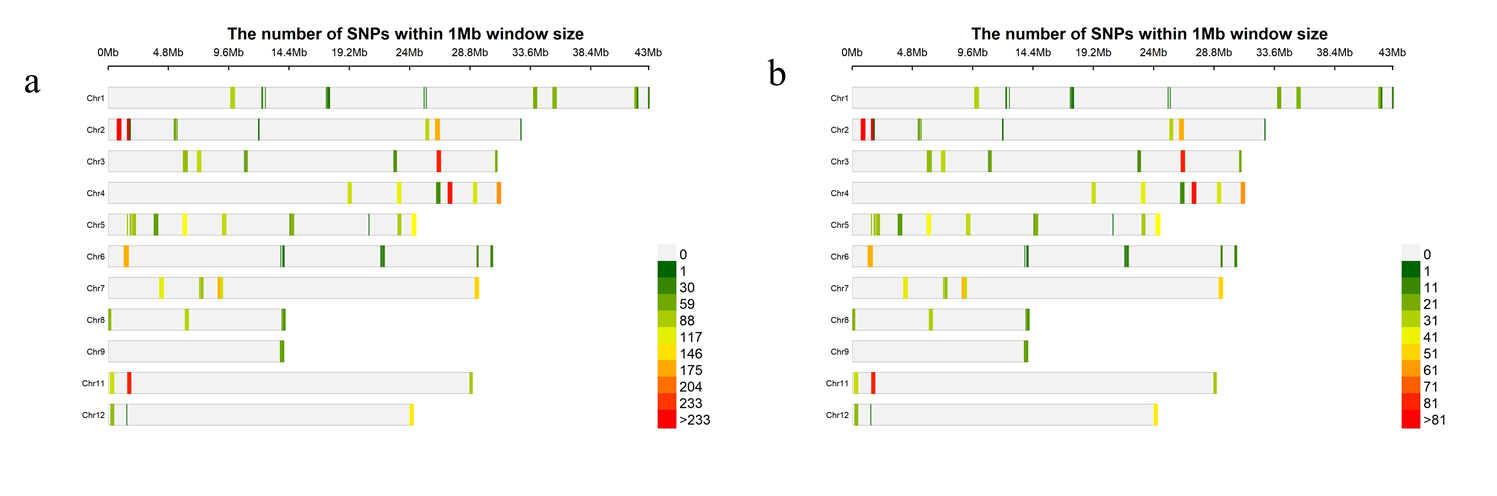


**Figure S2** Marker distribution in SEA, SC and DC1 panels.

**a**. SNP and InDels in SEA and SC panel. **b**. SNP in DC1 panel
